# Burden of disease attributable to unsafe drinking water, sanitation, and hygiene in domestic settings: a global analysis for selected adverse health outcomes

**DOI:** 10.1016/S0140-6736(23)00458-0

**Published:** 2023-06-17

**Authors:** Jennyfer Wolf, Richard B Johnston, Argaw Ambelu, Benjamin F Arnold, Robert Bain, Michael Brauer, Joe Brown, Bethany A Caruso, Thomas Clasen, John M Colford, Joanna Esteves Mills, Barbara Evans, Matthew C Freeman, Bruce Gordon, Gagandeep Kang, Claudio F Lanata, Kate O Medlicott, Annette Prüss-Ustün, Christopher Troeger, Sophie Boisson, Oliver Cumming

**Affiliations:** a Department of Environment, Climate Change and Health, World Health Organization, Geneva, Switzerland; b Division of Water and Health, Ethiopian Institution of Water Resources, Addis Ababa University, Addis Ababa, Ethiopia; c FI Proctor Foundation, University of California, San Francisco, CA, USA; d UNICEF Middle East and North Africa, Amman, Jordan; e Institute for Health Metrics and Evaluation, University of Washington, Seattle, WA, USA; f Department of Health Metrics Sciences, University of Washington, Seattle, WA, USA; g School of Population and Public Health, University of British Columbia, Vancouver, BC, Canada; h Department of Environmental Sciences and Engineering, Gillings School of Global Public Health, University of North Carolina at Chapel Hill, Chapel Hill, NC, USA; i The Hubert Department of Global Health, Rollins School of Public Health, Emory University, Atlanta, GA, USA; j Gangarose Department of Environmental Health, Rollins School of Public Health, Emory University, Atlanta, GA, USA; k Division of Epidemiology and Biostatistics, School of Public Health, University of California, Berkeley, CA, USA; l School of Civil Engineering, University of Leeds, Leeds, UK; m Wellcome Trust Research Laboratory, Division of Gastrointestinal Sciences, Christian Medical College, Vellore, Tami Nadu, India; n Instituto de Investigación Nutricional, Lima, Peru; o School of Medicine, Vanderbilt University, Nashville, TN, USA; p Department of Epidemiology, London School of Hygiene & Tropical Medicine, London, UK; q Department of Disease Control, Faculty of Infectious Tropical Disease, London School of Hygiene & Tropical Medicine, London, UK

## Abstract

**Background:**

Assessments of disease burden are important to inform national, regional, and global strategies and to guide investment. We aimed to estimate the drinking water, sanitation, and hygiene (WASH)-attributable burden of disease for diarrhoea, acute respiratory infections, undernutrition, and soil-transmitted helminthiasis, using the WASH service levels used to monitor the UN Sustainable Development Goals (SDGs) as counterfactual minimum risk-exposure levels.

**Methods:**

We assessed the WASH-attributable disease burden of the four health outcomes overall and disaggregated by region, age, and sex for the year 2019. We calculated WASH-attributable fractions of diarrhoea and acute respiratory infections by country using modelled WASH exposures and exposure–response relationships from two updated meta-analyses. We used the WHO and UNICEF Joint Monitoring Programme for Water Supply, Sanitation and Hygiene public database to estimate population exposure to different WASH service levels. WASH-attributable undernutrition was estimated by combining the population attributable fractions (PAF) of diarrhoea caused by unsafe WASH and the PAF of undernutrition caused by diarrhoea. Soil-transmitted helminthiasis was fully attributed to unsafe WASH.

**Findings:**

We estimate that 1·4 (95% CI 1·3–1·5) million deaths and 74 (68–80) million disability-adjusted life-years (DALYs) could have been prevented by safe WASH in 2019 across the four designated outcomes, representing 2·5% of global deaths and 2·9% of global DALYs from all causes. The proportion of diarrhoea that is attributable to unsafe WASH is 0·69 (0·65–0·72), 0·14 (0·13–0·17) for acute respiratory infections, and 0·10 (0·09–0·10) for undernutrition, and we assume that the entire disease burden from soil-transmitted helminthiasis was attributable to unsafe WASH.

**Interpretation:**

WASH-attributable burden of disease estimates based on the levels of service established under the SDG framework show that progress towards the internationally agreed goal of safely managed WASH services for all would yield major public-health returns.

**Funding:**

WHO and Foreign, Commonwealth & Development Office.

## Introduction

Despite substantial progress, unsafe drinking water, sanitation, and hygiene (WASH) services continue to pose important risks to health.^1^, ^2^ Billions of people do not use safely managed drinking water and sanitation services^3^ and hundreds of millions do not use even basic WASH services (panel). Unsafe WASH increases the risk of diarrhoea,^4^ chronic undernutrition because of repeated bouts of diarrhoea,^5^, ^6^ acute respiratory infections,^7^ and soil-transmitted helminthiasis.^8^ Although the evidence is scarce with regard to quantifying the exposure–response relationships, unsafe WASH is associated with various other adverse outcomes. Examples include: trachoma;^9^ schistosomiasis;^10^ hepatitis;^11^ conditions related to naturally occurring and synthetic chemical exposures such as arsenicosis, fluorosis, and lead poisoning;^11^ and longer term consequences such as childhood stunting.^12^ Additional pathways through which unsafe WASH might negatively affect health include inflammation and changes in the gut microbiome.^13^, ^14^ There is also evidence of compromised WASH conditions affecting educational outcomes, cognitive development, and wellbeing including mental health, contributing to bodily injury, and resulting in physical and sexual violence, particularly among women and girls.^15^, ^16^

Panel **Drinking water, sanitation, and hygiene (WASH) service levels3 and Sustainable Development Goal (SDG) WASH indicators**

**Drinking water service level**

• Safely managed: Drinking water from an improved source that is accessible on the premises, available when needed and free from faecal and priority chemical contamination
• Basic: Drinking water from an improved source, provided collection time is not more than 30 min for a round trip, including queuing
• Limited: Drinking water from an improved source, for which collection time exceeds 30 min for a round trip, including queuing
• Unimproved: Drinking water from an unprotected dug well or unprotected spring
• Surface water: Drinking water directly from a river, dam, lake, pond, stream, canal, or irrigation canal

Improved drinking water sources include piped water, boreholes or tubewells, protected dug wells, protected springs, rainwater, and packaged or delivered water.

**Sanitation service level**

• Safely managed: Use of improved facilities that are not shared with other households and where excreta are safely disposed of in situ or removed and treated offsite
• Basic: Use of improved facilities that are not shared with other households
• Limited: Use of improved facilities that are shared with other households
• Unimproved: Use of pit latrines without a slab or platform, hanging latrines, or bucket latrines
• Open defecation: Disposal of human faeces in fields, forests, bushes, open bodies of water, beaches or other open places, or with solid waste

Improved sanitation facilities include flush or pour flush toilets connected to piped sewer systems, septic tanks, or pit latrines; pit latrines with slabs (including ventilated pit latrines); and composting toilets.

**Hygiene service level**

• Basic: Availability of a handwashing facility with soap and water at home
• Limited: Availability of a handwashing facility lacking soap or water at home
• No facility: No handwashing facility at home

Handwashing facilities might be located within the dwelling, yard, or plot. They can be fixed or mobile and include a sink with tap water, buckets with taps, tippy-taps, and jugs or basins designated for handwashing. Soap includes bar soap, liquid soap, powder detergent, and soapy water but does not include ash, soil, sand, or other handwashing agents.

**SDG WASH targets and indicators**

6.1: By 2030, achieve universal and equitable access to safe and affordable drinking water for all

6.1.1: Proportion of population using safely managed drinking water services

6.2: By 2030, achieve access to adequate and equitable sanitation and hygiene for all and end open defecation, paying special attention to the needs of women and girls and those in vulnerable situations

6.2.1: proportion of population using (A) safely managed sanitation services and (B) a hand-washing facility with soap and water

Previous WASH-attributable burden of disease assessments have used comparative risk assessment methods for diarrhoea, acute respiratory infections,^1^, ^2^ and schistosomiasis^1^ and have also included other outcomes such as malaria, undernutrition, soil-transmitted helminthiasis, and trachoma using other methods that allowed more limited underlying exposure and exposure–response data.^1^ Including these outcomes, WHO estimated that 1·6 million deaths in 2016 could be attributed to unsafe WASH,^1^ whereas the Institute of Health Metrics and Evaluation (IHME) estimated that 1·7 million deaths in 2019 resulted from WASH-attributable diarrhoea and acute respiratory infections.^2^

**Research in context**

**Evidence before this study**

The disease burden attributable to unsafe drinking water, sanitation, and hygiene (WASH) has been estimated multiple times by different institutions, such as WHO and the Institute for Health Metrics and Evaluation (IHME). These analyses have used different counterfactuals ranging from basic WASH services to high-quality piped water with additional treatment, sewered sanitation systems or basic sanitation reaching high community coverage, and handwashing with soap with assigned exposure levels having low or no assumed disease risk. Previous WASH-attributable burden of disease assessments done by WHO and IHME have estimated the WASH-attributable burden for diarrhoea and acute respiratory infections. WHO's assessment also included WASH-attributable undernutrition, soil-transmitted helminthiasis, trachoma, malaria, and schistosomiasis, with a total disease burden of about 1·6 million deaths and over 100 million disability-adjusted life-years from unsafe WASH. When counterfactuals are set at the higher service levels as reflected by the Sustainable Development Goal (SDG) targets 6.1 and 6.2, the attributable burden of disease is larger. However, reliable exposure data for these higher levels of service, and epidemiological evidence of their effects on health outcomes, remain more difficult to obtain. Evidence for the exposure–response relationship between WASH exposures and diarrhoea, and WASH exposures and acute respiratory infections is taken from two current meta-analyses that include intervention studies about WASH improvements and these health outcomes.

**Added value of this study**

This study estimates the WASH-attributable burden of diarrhoea, acute respiratory infections, undernutrition, and soil-transmitted helminthiasis that can be prevented by meeting SDG targets 6.1 and 6.2. In contrast to previous estimates that were based on other minimum risk-exposure levels, this WASH-attributable disease assessment reflects levels of service established under the SDG framework. Although comprehensive data on SDG indicators are still scarce, our estimates show the additional value of collecting information on these service levels to reflect the full burden of disease associated with unsafe WASH.

**Implications of all the available evidence**

Although continued improvements in WASH are reducing the global burden of disease from diarrhoea, acute respiratory infections, undernutrition, and soil-transmitted helminthiasis, there are important health benefits that can be obtained in reaching SDG targets 6.1 and 6.2. Accurate estimates will require continued efforts to establish the effect of WASH on other diseases and wellbeing outcomes and to estimate exposure and exposure–response data for the SDG WASH indicators, especially safely managed drinking water and sanitation services.

In this study, we present burden of disease estimates attributable to unsafe WASH for the year 2019 for diarrhoea, acute respiratory infections, undernutrition, and soil-transmitted helminthiasis. Disease burden assessments raise awareness about the importance of different risk factors, translate scientific results into population-level estimates of health effects, and assist in setting priorities and choosing interventions with the largest expected public-health effect.^17^ This analysis estimates the WASH-attributable burden of disease based on the UN Sustainable Development Goal (SDG) targets for WASH,^18^ which are recognised as attainable policy goals (panel).^17^ These estimates are used to track progress towards SDG target 3.9, which calls upon countries to substantially reduce the number of deaths and illnesses from hazardous chemicals and air, water, and soil pollution and contamination by 2030. Our estimates can support efforts to improve use of safe WASH services and inform the recommendations of the ongoing Lancet *Commission on water, sanitation and hygiene, and health*.^19^

## Methods

### Study design

For this burden of disease assessment, unsafe WASH spans a range of use of drinking water, sanitation, and hygiene services and technologies and behaviours, which influence the risk for disease transmission. For grouping the population into exposure categories, we used different levels of WASH services using the terms unimproved, limited, basic, and safely managed as defined by the WHO and UNICEF Joint Monitoring Programme for Water Supply, Sanitation and Hygiene (JMP) for monitoring progress against SDG targets 6.1 and 6.2. We did not include disease burden assessment attributable to other risks such as unsafe water-resource management or unsafe water bodies.

For the assessment of the WASH-attributable burden of diarrhoea and acute respiratory infections we used the results of the most up-to-date evidence^4^, ^5^, ^6^, ^7^ on interventions improving WASH access, use, and related disease outcome.

We followed guidelines for accurate and transparent reporting (appendix 1 pp 15–16). Analyses were done with Stata (version 14).

Disease outcomes with a strong epidemiological link to unsafe WASH were included if they had sufficient data available to allow the quantification of the WASH-attributable burden preventable by improving WASH services. The WASH-attributable burden of diarrhoea and acute respiratory infections was estimated using comparative risk assessment. For undernutrition and soil-transmitted helminthiasis a standard comparative-risk-assessment approach was not possible because of a paucity of data on both exposure and the exposure–response relationship; we therefore used other methods (Table 1, Table 2). We produced disease burden estimates for males and females of all ages living in 183 WHO member states,^20^ representing 99·5% of the global population. Disease burden attributable to WASH was estimated for 27 low-income countries (LICs), 54 lower-middle-income countries (LMICs), and 51 upper-middle-income countries (UMICs; appendix 1 pp 1–2).^21^ As most of the 51 high-income countries (HICs) have near universal access to safely managed drinking water and sanitation services and as the available epidemiological evidence from intervention studies linking drinking water, sanitation, and disease outcomes originates mainly from LMICs,^4^ we do not estimate the burden of disease attributable to unsafe water and sanitation in HICs. However, handwashing with soap and water after toilet use is not universally practiced in HICs, and we do estimate disease burden from diarrhoea and acute respiratory infections due to inadequate hygiene in HICs.

**Table 1:** Counterfactual and outcome association for diarrhoea and acute respiratory infections

	Prevalence of WASH minimum risk exposure counterfactual in 2019*	**Association between WASH counterfactual and outcome (against lowest level of exposure)**
**Diarrhoea**		
Safely managed drinking water	37·9% (27·1–49·9)	0·48 (0·26–0·87), p=0·017^4^
Basic sanitation connected to sewer	29·7% (23·9–36·1)	0·53 (0·30–0·93), p=0·030^4^
Handwashing with soap after potential faecal contact	26·4% (23·4–29·6)	0·7 (0·64–0·76), p<0·0001^4^
**Acute respiratory infections**		
Handwashing with soap after potential faecal contact	26·4% (23·4–29·6)	0·83 (0·76–0·90), p<0·0001^7^

Data are prevalence (95% CI) or relative risk (95% CI), p value. WASH=drinking water, sanitation, and hygiene.

* Aggregated across included countries.

**Table 2:** Counterfactual and methods for undernutrition and soil-transmitted helminthiasis

	**WASH counterfactual exposure**	**Methods**	**Limitations**
Undernutrition	As for diarrhoea in table 1 (because based on WASH diarrhoea PAFs)	Combining the PAF of malnutrition attributable to diarrhoea with the PAF of diarrhoea attributable to unsafe WASH	Considers only one of multiple potential pathways linking unsafe WASH and undernutrition and therefore might represent only a fraction of WASH-attributable undernutrition
Soil-transmitted helminthiasis*	Safely managed drinking water, safely managed sanitation, and handwashing with soap	Complete attribution of overall disease burden estimates	Assumes that all soil-transmitted helminthiasis could be prevented through safe WASH

WASH=drinking water, sanitation, and hygiene. PAF=population attributable fraction.

* Ascaris lumbricoides, Trichuris trichiura, and hookworms.

Contrary to the previous WASH-attributable burden of disease assessment done by WHO,^1^ we did not include malaria as a health outcome, because the attribution was made to unsafe water resource management, rather than unsafe WASH. We also did not include schistosomiasis and trachoma, because the available exposure–response data relate to modest WASH improvements such as basic WASH services.^10^ Previous burden of disease assessments^22^ have estimated population attributable fractions (PAFs) for other vector-borne diseases such as dengue and lymphatic filariasis through structured expert surveys, a method which has been shown to have numerous limitations.^23^

Since these estimates of the WASH-attributable burden of disease are used to track SDG 3.9, a consultation with national authorities was run by WHO from April 5, 2022, to June 30, 2022, to seek feedback on estimates before finalisation.

### Comparative risk assessment

**Figure 1:**
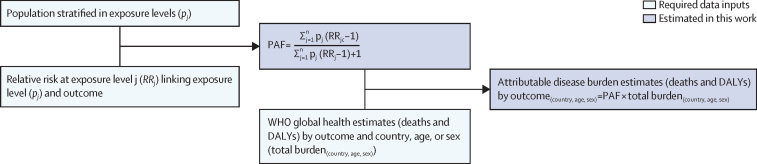
General approach for calculating the PAF and the attributable disease burden in comparative risk assessment Sources for data inputs are listed in appendix 1 (p 6). DALY=disability-adjusted life-year. PAF=population attributable fraction. n=total number of exposure levels.

**Figure 2:**
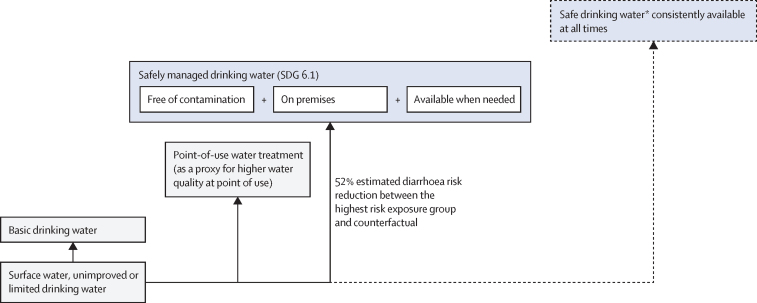
Conceptual model for disease burden assessment attributable to unsafe drinking water and associated reduction in risk of diarrhoea^4^ Blue box with solid line: counterfactual minimum risk exposure used in the analysis with associated percentage reduction in risk of diarrhoea compared with the highest risk-exposure group from a meta-analysis.^4^ Blue box with dashed line: additional plausible risk reduction, which cannot currently be estimated due to a paucity of exposure or exposure–response data. SDG=Sustainable Development Goal. *Safe drinking water does not represent any substantial risk to health over a lifetime of consumption. The position of the boxes is not directly proportional to the expected health effect.

**Figure 3:**
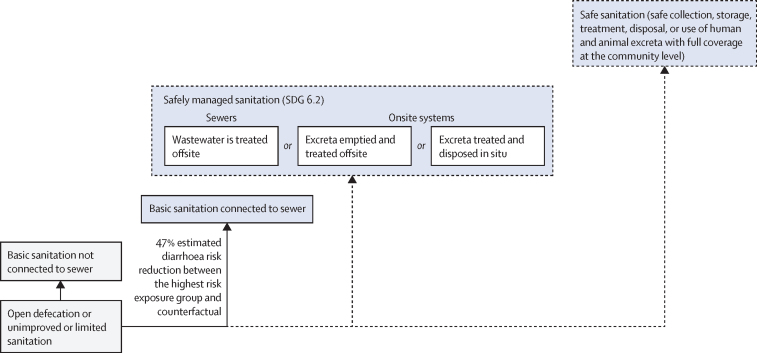
Conceptual model for disease burden assessment attributable to unsafe sanitation and associated reduction in risk of diarrhoea^4^ Blue box with solid line: counterfactual minimum risk exposure used in the analysis with associated percentage reduction in risk of diarrhoea compared with the highest risk-exposure group from Wolf and colleagues’^4^ meta-analysis. Blue boxes with dashed lines: additional plausible risk reduction, which cannot currently be estimated due to a paucity of exposure or exposure–response data. The position of the boxes is not directly proportional to the expected health effect. SDG=Sustainable Development Goal.

**Figure 4:**
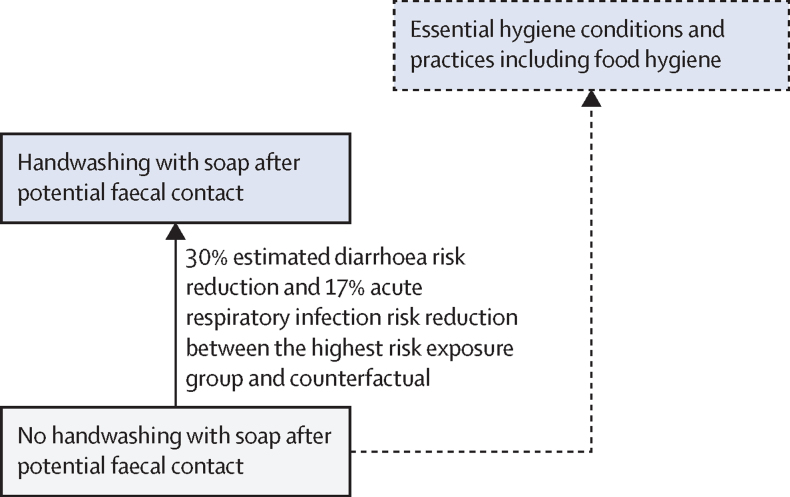
Conceptual model for disease burden assessment attributable to unsafe hygiene and associated reductions in risk of diarrhoea and acute respiratory infections^4^, ^7^ Blue box with solid line: counterfactual minimum risk exposure used in the analysis with associated percentage reductions in risk of diarrhoea^4^ and risk of acute respiratory infections^7^ compared with no handwashing. Blue box with dashed line: additional plausible risk reductions that cannot currently be estimated due to a paucity of exposure or exposure–response data. The position of the boxes is not directly proportional to the expected health effect.

The standard approach for estimating the burden of disease attributable to a given risk factor is comparative risk assessment (figure 1).^24^, ^25^ Comparative risk assessments systematically evaluate changes in population health as a consequence of changing the distribution of a risk factor's exposure in the population.^26^ The approach requires the distribution of population's exposure (*p*) to the relevant risk factor levels (j) and the exposure–response relationships (relative risk [RR]) between different exposure levels (p_j_) and the health outcome, usually based on a pooled analysis of high quality interventions (appendix 1 p 10).^27^ A counterfactual minimum exposure level—corresponding to the removal or reduction of exposure—is specified and compared with the current distribution of the risk factor's exposure in the population of interest. For this analysis, we used counterfactual minimum risk-exposure levels that aligned as closely as possible with the SDG indicators (panel, Figure 2, Figure 3, Figure 4), to the extent that available exposure and exposure–response data permitted.

### Population exposed (p_j_)

To estimate the population exposure to different WASH service levels (figure 1), we used the public database of the JMP, which draws upon nationally representative surveys, censuses, and administrative data to produce national, regional, and global estimates of WASH service use. The JMP extracts data from national data sources and matches them to global indicators and definitions, to the extent possible, to ensure comparability and consistency. These harmonised extracts are used as inputs to a regression model to produce draft estimates, which undergo a country consultation as an additional quality-control measure before finalisation.

As some countries did not have sufficient national data, we applied multilevel modelling to estimate the use of different WASH services and household water treatment for 2019 using a two-level random slope model for use of different drinking water and sanitation services and a two-level random intercept model for use of household water treatment and access to basic handwashing facilities. For countries without datapoints, we used the regional mean estimate of the model. Further details on input data and the modelling approach have previously been published (appendix 1 pp 2–3).^28^

We adjusted estimates of safely managed drinking water services to incorporate nationally representative survey data on the proportion of households meeting all three criteria for safely managed drinking water (quality, availability, and accessibility) at the household level.^29^ This adjustment was made because JMP estimates of safely managed drinking water assess microbiological water quality at the point of collection, not at the point of consumption, and therefore do not account for the possibility of contamination after collection;^30^ and, due to the paucity of data, these estimates are based on the minimum of quality, availability, and accessibility at urban and rural levels for each country, rather than the proportion of the population meeting all three criteria at the household level (appendix 1 pp 4–5).

For sanitation, we would ideally have chosen a counterfactual of safely managed sanitation; however, this was not possible due to the current paucity of data on both exposure and exposure–response. Instead, using the latest epidemiological evidence,^4^ we used a counterfactual of basic sanitation connected to sewer networks (figure 3), which is not necessarily equivalent to safely managed sanitation as much of the wastewater collected in sewers is not safely treated.^31^

Estimates for access to basic handwashing facilities at home do not necessarily reflect the actual practice of handwashing with soap, since many people do not wash hands after toilet use even if soap and water are available.^32^ We therefore adjusted these estimates based on the results of a meta-analysis of the association between presence of a handwashing station with soap and water and observed handwashing practices.^33^

### Relative risks linking exposure and health outcome (RR_j_)

The exposure–response relationships (figure 1, appendix 1 p 6) linking the different WASH exposure levels (Figure 2, Figure 3, Figure 4) and diarrhoea or acute respiratory infections have been estimated in two meta-analyses.^4^, ^7^ For drinking water, the highest diarrhoea-risk reduction relates to improved drinking water on premises with higher water quality, for sanitation to basic sanitation connected to sewer, and for hygiene to handwashing promotion usually with the provision of hygiene infrastructure such as handwashing stations with water and soap (Table 1, Table 2).^4^

### PAF and attributable burden estimates

The PAF is the proportion of total morbidity or mortality due to a condition or disease that could have been prevented by reducing the risk factor to a counterfactual defined by the minimum risk-exposure level.^27^ The PAF is estimated through combining population exposure and corresponding relative risks (figure 1). To estimate the risk factor-attributable burden, the PAF is multiplied with the total disease burden. We use WHO global health estimates (disability-adjusted life-years [DALYs] and deaths) for the included health outcomes by country, sex, and age groups in 2019 (appendix 2 pp 3–6).^34^, ^35^ Tables with included International Classification of Diseases-10 codes are listed in appendix 1 (pp 6–8). Further information on the preparation of WHO Global Health Estimates is provided in two technical reports.^36^, ^37^

Disease burden can be caused by different risks. Although the PAF for each risk factor, such as unsafe WASH, is a proportion and is bounded by 0 and 1, the sum of the PAFs from the individual relevant exposures can exceed 1.^38^ For example, attributing 100% of soil-transmitted helminthiasis to unsafe WASH does not preclude some proportion of this WASH-attributable disease burden being eliminated through other interventions, such as deworming medication.

Estimation of uncertainty intervals at the country, regional, and global levels was done with Monte Carlo simulation (appendix 1 p 5).

### Diarrhoea and acute respiratory infections

Diarrhoeagenic pathogens are transmitted via previously described environmental routes,^39^ which can be interrupted with safe WASH. Respiratory pathogens are transmitted through the air, person-to-person contact, or via surfaces, and handwashing with soap can remove or destroy pathogens on hands, thereby reducing transmission.^7^ The burden of diarrhoea attributable to unsafe WASH and the burden of acute respiratory infections attributable to unsafe hygiene are estimated using comparative risk assessment (figure 1). We used the following standard formula

$${\text{PAF}}_{\text{WASH}}=1-\overset{\text{R}}{\underset{\text{r}=\text{1}}{\Pi }}\left(1-{\text{PAF}}_{\text{r}}\right)$$

to estimate the burden of diarrhoea attributable to the risk factor cluster of unsafe WASH combined:^40^ where r is the individual risk factor (unsafe drinking water, sanitation, or hygiene), and R is the total number of risk factors (three) accounted for in the cluster.

### Undernutrition

For calculating the WASH-attributable burden of undernutrition, we did not use a comparative risk assessment as the available exposure–response relationships from systematic reviews and meta-analyses relate to modest WASH improvements such as household water treatment, basic drinking water, sanitation, or hygiene provision, and hygiene education alone or in combination^12^, ^41^, ^42^, ^43^ and do not reach full safety. We followed a previously published approach (appendix 1 p 5).^1^ Undernutrition can be a consequence of repeated bouts of diarrhoea.^5^, ^13^ We multiplied country-level PAFs of diarrhoea attributable to unsafe WASH as estimated in this study with previously published PAFs of protein-energy malnutrition attributable to diarrhoea (appendix 2 p 1).^6^ In the Global Burden of Diseases, Injuries, and Risk Factors Study (GBD),^6^ protein–energy malnutrition is exclusively considered an outcome of childhood underweight and wasting. Troeger and colleagues^6^ estimated the attributable fractions for protein–energy malnutrition attributable to underweight and wasting independently from a counterfactual model that quantified the expected shift in the distribution of weight-for-age and weight-for-height in the absence of diarrhoea compared with the observed distributions.^44^ We multiplied the resulting PAF of undernutrition attributable to unsafe WASH with WHO total disease burden figures for protein energy malnutrition for children younger than 5 years (appendix 1 p 5).

### Soil-transmitted helminthiasis

This assessment includes infections with the major soil-transmitted helminths that infect humans: *Ascaris lumbricoides*, *Trichuris trichiura*, and hookworms (*Necator americanus* and *Ancylostoma duodenale*).

Soil-transmitted helminths are transmitted by eggs present in human faeces of an infected individual, which then enter soil in the absence of safe sanitation. Infection occurs through ingestion of eggs attached to vegetables, in contaminated water sources or soil, or—by hookworms—active penetration of skin by larvae. There is no direct person-to-person transmission or infection from fresh faeces as the excreted eggs require 5–10 days to mature in the soil before becoming infective. *A lumbricoides, T trichiura,* and hookworms do not multiply in the human host and re-infection only occurs as a result of contact with infective stages in the environment.^45^

Based on these requirements for transmission, we assumed that transmission would be interrupted if everyone used safely managed WASH services and practiced handwashing with soap after potential faecal contact.

Sources of exposure, exposure–response, and overall disease burden data are listed in appendix 1 (p 6).

### Role of the funding source

The funders had no role in study design, data collection, data analysis, data interpretation, writing of the manuscript, or the decision to submit the manuscript for publication.

## Results

After adjusting the estimates for safely managed drinking water, 37·9% (95% CI 29·1–49·9) of the population in LMICs used safely managed drinking water (calculated at the household level), 29·7% (23·9–36·1) used basic sanitation connected to sewer networks, and 26·4% (23·4–29·6) of the global population washed hands with soap after potential faecal contact. Estimates of WASH exposure levels and matching exposure–response relationships are shown in Table 1, Table 2 and regional aggregates are in appendix 1 (pp 8–10). Country-level exposure estimates derived through multilevel modelling for this analysis and official exposure estimates from the global SDG database maintained by the JMP are available in appendix 2 (pp 7–9).

The WASH-attributable disease burden combined across the four outcomes amounts to 1 401 000 deaths (95% CI 1 283 000–1 542 000) and 73 935 000 DALYs (68 248 000–80 186 000) in 2019. An estimated 273 000 (252 000–296 000) deaths from diarrhoea among children younger than 5 years were attributable to unsafe WASH in 2019. Additionally, 112 000 (92 000–134 000) children younger than 5 years died from acute respiratory infections attributable to unsafe hygiene in 2019 (appendix 2 pp 2–4).

We estimate that 69% of diarrhoea, 14% of acute respiratory infections, and 10% of undernutrition, and assume 100% of the burden of soil-transmitted helminthiasis, could be have been prevented with safe WASH in 2019 (table 3). There are considerable differences in the WASH-attributable disease burden between income groups: there were 270 000 deaths in LICs, 975 000 deaths in LMICs, and 112 000 deaths in UMICs, compared with 44 000 deaths in HICs (though deaths from unsafe water and sanitation were not estimated for HICs). There is also substantial regional variation ranging from 510 000 WASH-attributable deaths in WHO's Africa region and 593 000 deaths in WHO's South-East Asia region to 33 000 deaths in WHO's European region. Furthermore, we found substantial variation in the WASH-attributable fraction of the different diseases. For example, 18% of the diarrhoea-related disease burden in HICs could be prevented through safe WASH compared with 76% in LMICs in WHO's Africa region and 66% in LMICs in WHO's South-East Asia region (appendix 1 p 12).

**Table 3:** WASH-attributable disease burden by health outcome, 2019

	**PAF (95% CI)**	**Deaths (95% CI)**	**DALYs (95% CI)**
Diarrhoea	0·69 (0·65–0·72)	1 035 000 (929 000–1 160 000)	54 590 000 (50 033 000–59 562 000)
Acute respiratory infections	0·14 (0·13–0·17)	356 000 (320 000–405 000)	16 578 000 (14 257 000–19 481 000)
Undernutrition	0·10 (0·09–0·10)	8000 (7000–9000)	825 000 (755 000–905 000)
Soil-transmitted helminthiasis*	1·0†	2000 (2000–3000)	1 942 000 (1 862 000–2 028 000)

WASH=drinking water, sanitation, and hygiene. PAF=population attributable fraction. DALY=disability-adjusted life-year.

* *Ascaris lumbricoides*, *Trichuris trichiura,* and hookworms*.*

† Assumed value.

## Discussion

We estimate that 1·4 million deaths and 74 million DALYs could have been prevented through the universal provision of safe WASH in 2019, accounting for 2·5% of all deaths and 2·9% of all DALYs in the global population and 7·6% of all deaths and 7·5% of all DALYs in children younger than 5 years. Diarrhoea accounts for the majority of the WASH-attributable burden with over 1 million deaths, about 55 million DALYs, and a preventable fraction of 69%, followed by acute respiratory infections attributable to unsafe hand hygiene with about 356 000 deaths, 17 million DALYs, and a preventable fraction of 14%.

Our estimates are lower than those from GBD 2019,^2^ which estimated 1·7 million deaths and 88 million DALYs. Differences arise from the expanded attributable health outcomes from more up-to-date epidemiological evidence and, especially, the adoption of different counterfactual minimum risk-exposure levels. GBD 2019^2^ used high-quality piped water that is boiled or filtered at point of use as the minimum risk-exposure level for drinking water. To estimate this exposure level, piped water was divided into basic-quality and high-quality piped water based on the results of a systematic review done in 2013, which included a mixture of private and community piped drinking-water services and predominantly measured drinking water quality at one point in time.^46^ On the exposure–response side, GBD 2019 used a pooled RR of 0·09 (corresponding to a 91% reduction in the risk of diarrhoea for drinking water improvements alone) by multiplying the effect of filtering or boiling at the household level with the effect of providing high-quality piped water.^47^ The epidemiological studies providing exposure–response relationships for point-of-use interventions are usually done in settings with a high level of unimproved drinking water sources, which calls into question the approach of multiplying the effect of point-of-use interventions with the effect of providing high-quality piped water. Additionally, point-of-use interventions often require intensive supervision and follow-up to sustain water treatment, which could lead to incorrectly estimated health effects from reporting bias. A comparison of WHO and IHME WASH-attributable disease burden and minimum risk-exposure levels over time is included in appendix 1 (p 13).

This analysis expands upon previous minimum risk-exposure levels by using the SDG ambition of universal access to safely managed WASH services as minimum risk-exposure levels. We consider these levels more attainable than a theoretical zero-risk counterfactual because they reflect the ambition set out in the internationally agreed SDGs and have already been achieved by numerous countries across different regions of the world. In addition, nationally representative exposure data on safely managed services is available for an increasing number of countries aggregated and published by the JMP and is combined with matching exposure–response relationships available through the up-to-date meta-analyses.^4^, ^7^ Our results, therefore, offer a policy-relevant complement to previous analyses and might influence the choice of future counterfactual minimum risk exposures in evaluations of health effects of other risk factors included under the SDGs.

Our analysis has important limitations. Due to insufficient data, we excluded multiple potentially important health outcomes, which makes it probable that we underestimated the true burden. We restricted our estimation of disease burden attributable to WASH to the four health outcomes for which there was sufficient information to estimate the exposure–response relationship (ie, intervention studies that estimated the health effect for higher levels of WASH services), PAFs that could be taken from the published literature, or certain requirements in the disease-transmission pathway that allowed the assumption of attributing the total disease burden of a certain disease—here, soil-transmitted helminthiasis—to unsafe WASH. A list of other adverse health outcomes linked to unsafe WASH or related exposures, including chemical exposures, is included in appendix 1 (pp 14–15). We estimated on an exploratory basis the additional attributable disease burden had we considered other outcomes for which a PAF has been estimated in previous analyses, including trachoma, schistosomiasis, malaria, lymphatic filariasis, onchocerciasis, and dengue (appendix 1 p 14). We multiplied these PAFs with overall disease burden estimates by outcome and for the year 2019. From this result, we estimated an additional 360 000 attributable deaths and 31 million DALYs. Of these, 99% of deaths and 97% of DALYs were from malaria, dengue, onchocerciasis, and lymphatic filariasis for which the risk factor was unsafe water resource management, which concerns very different interventions and strategies as compared with safe WASH service provision. Schistosomiasis and trachoma, the two outcomes related to unsafe WASH, add only about 5000 additional deaths and 900 000 DALYs—a very small proportion of the WASH-attributable disease burden estimated in this work. As for both trachoma and schistosomiasis we were not able to establish a counterfactual of higher level WASH service provision (ie, extending beyond basic WASH services) so we did not include their WASH-attributable burden of disease assessment in this analysis.

However, unsafe WASH affects health in many more ways: a high burden of paediatric asymptomatic carriage of certain bacteria and parasites due to unsafe WASH is strongly associated with adverse consequences, including growth faltering,^48^ and new estimates for the burden of disease attributable to antimicrobial resistance highlight the importance of community-based WASH to prevent these infections.^49^ WASH can affect other non-infectious-disease health outcomes—including physical and sexual trauma from violence, bodily injury such as from water carriage, mental health, and general wellbeing—particularly among women and girls.^15^, ^50^, ^51^ More evidence is needed to understand these additional outcomes and to generate sufficient data to enable their inclusion in future analyses.

In addition, our analysis is confined to domestic settings, so does not include the disease burden attributable to WASH in other settings such as schools, health-care facilities, workplaces, detention centres, refugee camps, markets, and other public spaces.

There are further limitations relating to the methods and data for the outcomes that we did include. For diarrhoea and acute respiratory infections we were able to use a standard comparative risk assessment approach based on systematic reviews that include a large number of rigorous studies. However, most of the underlying studies are open-label trials with subjective health outcomes (eg, self-reported diarrhoea or respiratory symptoms), so there is a risk of overestimating their effectiveness.^52^ In addition, many interventions were tightly controlled studies and the transportability of their results to population-level changes in WASH conditions remains uncertain.

For undernutrition and soil-transmitted helminthiasis, we judged there to be insufficient data for a comparative risk assessment approach and adopted alternative methods. For soil-transmitted helminthiasis, we attributed the complete disease burden to unsafe WASH based on knowledge about the disease transmission pathway. For undernutrition, we used published PAFs of protein–energy malnutrition from diarrhoea and combined them with the PAFs of diarrhoea from unsafe WASH. This two-step approach quantified only the WASH-attributable burden of protein–energy malnutrition that is a consequence of diarrhoea. There might be multiple additional mechanisms by which WASH could contribute to undernutrition, such as via parasitic infections,^53^ the asymptomatic carriage of certain enteric pathogens,^48^ and environmental enteric dysfunction.^12^, ^54^

Our results reflect the selected counterfactuals and assume no residual attributable disease burden where the complete population has met the SDG targets. However, there remains a considerable WASH preventable fraction of disease in countries with universal or near universal access to safely managed water,^55^ meaning that further improvements would yield further gains. Additionally, there remain many millions of people across HICs who do not have safe WASH but who are not adequately captured in official data.^56^ Almost a million people living in cities in the USA have been estimated to be without access to basic sanitation^57^ and nearly half a million households have been estimated to not have complete piped water services.^58^ Drinking water and sanitation-attributable diarrhoea burden in HICs might add several million additional DALYs to our estimates, but due to the paucity of data—especially on the exposure–response side—we do not include them in current estimates. In populations that have not achieved SDG targets for safely managed WASH services, our results further motivate complementary interventions that reduce the WASH-attributable disease burden, such as anthelmintics to treat soil-transmitted helminthiasis,^59^ and small-quantity nutritional supplementation to complement breastfeeding.^60^

Our results also reflect the preventable fraction of disease attributable to WASH systems functioning under current climate conditions. Global warming might alter the incidence or severity of several WASH preventable infectious diseases.^61^ Drought, flooding, and changes in water quality can interfere with water supply systems, resulting in intermittency and systems breakdown.^62^ Both higher intensity of rainfall and drought can reduce the operational effectiveness of sanitation systems leading to localised failure.^63^ Both effects could reduce the total prevented disease burden for populations with safe WASH.

Our classification of exposure to different WASH service levels relies on nationally representative survey data and administrative data, which might not accurately capture all aspects of safely managed services and have considerable gaps for higher levels of service, including for some populous countries. To estimate exposure to safely managed drinking water, we adjusted estimates using nationally representative survey data to account for the probability of water not contaminated at source being contaminated at the point of consumption and to assess the proportion of households meeting all three criteria (ie, accessibility, availability, and quality). For hygiene, we account for the probability of handwashing with soap occurring after faecal contact conditional on presence of handwashing facilities.^33^ These adjustments introduce uncertainty and do not adequately account for other important aspects of safely managed services, for example that drinking water is free from priority chemicals, such as arsenic and fluoride. The minimum risk-exposure level for sanitation is basic sanitation connected to sewer networks. This selection is based on a 2022 systematic review and meta-analysis^4^ that showed a lower RR compared with basic sanitation without sewered connections. Basic santitation connected to sewer networks do not assess all aspects of safely managed sanitation, such as the extent of wastewater treatment in centralised plants,^31^ and excludes other forms of safely managed sanitation (eg, onsite technologies where waste is emptied and treated off-site or safely disposed in situ). For both sanitation and water, there remains a paucity of rigorous epidemiological research to quantify the health effects of different types of safely managed WASH services. Due to the scarcity of the available data, there is also a risk of misclassification or absence of data for safely managed water and sanitation services that will hopefully reduce over time as countries adapt national monitoring systems for the higher service levels called for in the SDG framework.

We considered the regional distribution of the WASH-attributable disease burden, by the income level of countries, and by sex and age. Our findings of substantial variation by income status are supported by previous analyses^1^, ^64^ that found that the WASH attributable disease burden is highest in LMICs, and that the proportion of high-burden diseases, such as diarrhoea and acute respiratory infections, in these countries is much higher. Due to the paucity of data, we assume the same exposure and exposure–response relationship and therefore the same PAFs between WASH and disease outcomes for males and females, despite extensive research documenting that women and men have vastly different WASH experiences. Though we present WASH-attributable burden of disease estimates for males and females at the country level, these vary only due to differences in the overall disease burden figures. Women and girls might have increased exposures based on gendered expectations that they collect and treat water, manage dependents’ faeces, and care for ill children.^65^, ^66^, ^67^ Furthermore, all genders simultaneously have multiple intersecting identities (eg, race, socioeconomic status, and ability) that can influence exposure and exposure–response relationships related to WASH.^51^ The same issues relate to differential disease burden in different age groups.

For estimates that are closer to the true attributable burden of disease, better data are needed to more accurately characterise the population exposure to safely managed service levels and to quantify the exposure–risk relationship between WASH services, particularly high service levels including all aspects of safely managed WASH and different service types, and a broader range of health outcomes. Future research should be done in all, including high-income, settings.

Further research is also needed to better understand differences in exposure and exposure–response relationships between different sex and age groups to identify disparities in access to safe WASH services that are responsible for persistent inequalities.

As new data become available, future estimates might consider lower minimum risk-exposure levels, even extending safely managed WASH services by including, for example, continuously available drinking water free from microbial and chemical contamination, safe sanitation systems that extend to animal waste, and hygiene practices that extend beyond handwashing after faecal contact to including food hygiene, ideally in a combined safe WASH counterfactual exposure scenario.^68^

Combining up-to-date epidemiological evidence with exposures based as closely as possible on the levels of service established under the SDG framework, we estimate that meeting the WASH targets of SDG 6 could prevent at least 1·4 million deaths and 74 million DALYs annually. Although comprehensive data on SDG indicators are still scarce, our estimates show the additional value of collecting information on these service levels, particularly in low-resource settings, to accurately reflect the full burden of disease associated with WASH. As the UN report the need to accelerate progress towards the goal of universal access to safe WASH, our study shows the substantial health gains that can be obtained by redoubling efforts to achieve the SDG WASH targets.

## Data sharing

All data collected for this analysis including exposure, exposure–response data, overall disease statistics, and analytic code are available immediately following publication without end date to anyone for any purpose and is either published as supplementary material or can be accessed through the corresponding author.

## Declaration of interests

JW reports grants from UK Foreign, Commonwealth & Development Office (FCDO), during this study. MB reports grants from Bill & Melinda Gates Foundation during this study. CFL reports non-financial support from WHO COVID-19 Vaccine Effectiveness Working Group, grants from CureVac AG, grants from PATH, grants from HilleVax, and personal fees from Valneva outside the submitted work. JB reports grants from Bill & Melinda Gates Foundation, grants from US Centers for Disease Control & Prevention, and grants from Columbia World Projects during this study. MCF reports Consulting for Rickett's Global Scientific Advisory Committee. RBJ reports grants from UK FCDO during this study, grants from Agence française de développement, grants from Australian Government Department of Foreign Affairs and Trade, grants from Austrian Development Agency, grants from Bill & Melinda Gates Foundation, grants from German Federal Ministry for Economic Development and Cooperation, grants from The Netherlands Directorate-General for International Cooperation, grants from Swiss Agency for Development and Cooperation, and grants from United States Agency for International Development outside the submitted work. All other authors declare no competing interests.
