# Supplementary material for: Burden of disease attributable to unsafe drinking water, sanitation, and hygiene in domestic settings: a global analysis for selected adverse health outcomes
Source: Lancet. 2023 Jun 17;401(10393):2060–71. doi: 10.1016/S0140-6736(23)00458-0 (PMC10290941; doi:10.1016/S0140-6736(23)00458-0)
Supplement: Supplementary appendix 1 [file mmc1.pdf]

THE LANCET

Supplementary appendix

This appendix formed part of the original submission and has been peer reviewed. We post it as supplied by the authors.

Supplement to: Wolf J, Johnston RB, Ambelu A, et al. Burden of disease attributable to unsafe drinking water, sanitation, and hygiene in domestic settings: a global analysis for selected adverse health outcomes. *Lancet* 2023; published online June 5. https://doi.org/10.1016/S0140-6736(23)00458-0.

Appendix 1

Table of contents

1. WHO regional groupings and country list 1
2. Multilevel modeling of population exposures to different levels of WASH services 2
3. Estimation of safely managed drinking water adjusted for household level and point of use quality 4
5. WASH-attributable burden of malnutrition 5
6. Sources of exposure, exposure-response and disease data by outcome 6
7. Exposure estimates 8
8. Details on calculating attributable disease burden estimates following Figure 1 of the main manuscript 10
9. Regional estimates for WASH-attributable diarrhoea and ARI 10
10. Comparison of WASH-attributable disease burden between WHO and the IHME Global Burden of Disease (GBD) 13
11. List of diseases/adverse health outcomes that are linked to inadequate WASH or related risks 14
12. Gather checklist 15
13. Participants of the initial expert meeting on WASH-attributable disease burden estimates 16

References 17

1. WHO regional groupings and country list

WHO African Region: 47

Algeria, Angola, Benin, Botswana, Burkina Faso, Burundi, Cabo Verde, Cameroon, Central African Republic, Chad, Comoros, Congo, Côte d'Ivoire, Democratic Republic of the Congo, Equatorial Guinea, Eritrea, Eswatini, Ethiopia, Gabon, Gambia, Ghana, Guinea, Guinea-Bissau, Kenya, Lesotho, Liberia, Madagascar, Malawi, Mali, Mauritania, Mauritius, Mozambique, Namibia, Niger, Nigeria, Rwanda, Sao Tome and Principe, Senegal, Seychelles*, Sierra Leone, South Africa, South Sudan, Togo, Uganda, United Republic of Tanzania, Zambia, Zimbabwe.

WHO Region of the Americas: 35

Antigua and Barbuda*, Argentina, Bahamas*, Barbados*, Belize, Bolivia (Plurinational State of), Brazil, Canada*, Chile*, Colombia, Costa Rica, Cuba, {Dominica}, Dominican Republic, Ecuador, El Salvador, Grenada, Guatemala, Guyana, Haiti, Honduras, Jamaica, Mexico, Nicaragua, Panama, Paraguay, Peru, {Saint Kitts and Nevis*}, Saint Lucia, Saint Vincent and the Grenadines, Suriname, Trinidad and Tobago*, United States of America*, Uruguay*, Venezuela (Bolivarian Republic of).

WHO South-East Asia Region: 11

Bangladesh, Bhutan, Democratic People's Republic of Korea, India, Indonesia, Maldives, Myanmar, Nepal, Sri Lanka, Thailand, Timor-Leste.

WHO European Region: 53

Albania, {Andorra*}, Armenia, Austria*, Azerbaijan, Belarus, Belgium*, Bosnia and Herzegovina, Bulgaria, Croatia*, Cyprus*, Czechia*, Denmark*, Estonia*, Finland*, France*, Georgia, Germany*, Greece*, Hungary*, Iceland*, Ireland*, Israel*, Italy*, Kazakhstan, Kyrgyzstan, Latvia*, Lithuania*, Luxembourg*, Malta*, {Monaco*}, Montenegro, Netherlands*, North Macedonia, Norway*, Poland*, Portugal*, Republic of Moldova, Romania, Russian Federation, {San Marino*}, Serbia, Slovakia*, Slovenia*, Spain*, Sweden*, Switzerland*, Tajikistan, Türkiye, Turkmenistan, Ukraine, United Kingdom of Great Britain and Northern Ireland*, Uzbekistan.

WHO Eastern Mediterranean Region: 21

Afghanistan, Bahrain*, Djibouti, Egypt, Iran (Islamic Republic of), Iraq, Jordan, Kuwait*, Lebanon, Libya, Morocco, Oman*, Pakistan, Qatar*, Saudi Arabia*, Somalia, Sudan, Syrian Arab Republic, Tunisia, United Arab Emirates*, Yemen.

WHO Western Pacific Region: 27

Australia*, Brunei Darussalam*, Cambodia, China, {Cook Islands}, Fiji, Japan*, Kiribati, Lao People's Democratic Republic, Malaysia, {Marshall Islands}, Micronesia (Federated States of), Mongolia, {Nauru*}, New Zealand*, {Niue}, {Palau*}, Papua New Guinea, Philippines, Republic of Korea*, Samoa, Singapore*, Solomon Islands, Tonga, {Tuvalu}, Vanuatu, Viet Nam

* high-income (57)[1], countries in curly brackets indicate countries for which no total disease burden figures are available through the WHO Global Health Observatory (GHO)[2]

2. Multilevel modeling of population exposures to different levels of WASH services

Table S.1: Number of countries covered with ≥1 national data point and number of data points by WASH exposure category

	Number of countries (urban/rural)#	Number of data points (urban/rural)
**Drinking water**		
Improved water source	170/167	1839/1803
Water source within 30 minutes	140/139	609/608
Water source accessible on premises	169/166	787/774
Water available when needed	107/91	357/247
Water free from contamination	82/65	266/166
POU chlorination	103/103	190/190*
POU solar treatment	102/102	190/190*
POU filtration/boiling	103/103	190/190*
**Sanitation**		
Improved sanitation facility	169/166	1863/1854
Shared sanitation	155/153	759/752
Sanitation connected to sewer	155/152	1290/1277
**Hygiene**		
Basic handwashing facility with water and soap at home	98/99	193/199

POU: point of use; # out of 195 countries (WHO member states and territories), *includes POU treatment on different water exposures categories (e.g. POU chlorination on surface water, unimproved and limited drinking water, POU chlorination on improved, not on premises, POU chlorination, on premises)

Random slope model

This model is used for different drinking water and sanitation services.

$$\text{logit}(Y_{ij}) = \beta_0 + \beta_1 X_{1ij} + \sum_{k=2}^{18} \beta_k X_{kj} + \mu_j + u_{ij} X_{1ij} + \varepsilon_{ij}$$

where 'logit(Y_{ij})' is the proportion of use of a specific drinking water or sanitation service on the logit scale for year *i* and country *j*, 'β_0' is the overall mean intercept of Y across countries, 'β_1' is the regression coefficient for the predictor year X_{1ij} (defined at level 1: individual observations-level), 'β_k' are the regression coefficients for the regional covariates ('X_{kj}' – 17 regions), 'μ_j' are the residuals on the country level (group-level residuals), '$u_{ij}X_{1ij}$' is the interaction between country and year and 'ε_{ij}' are the residuals at the level of the observations (individual-level residuals).

Regional grouping for modelling of drinking water and sanitation services follow m49 classification and are divided as: Australia and New Zealand; Central Asia; Eastern Asia; Eastern Europe; Latin America and the Caribbean; Melanesia; Micronesia; Northern Africa; Northern America; Northern Europe; Polynesia; South-eastern Asia; Southern Asia; Southern Europe; Sub-Saharan Africa; Western Asia; Western Europe.

Random intercept model

Random intercept models are simpler compared to random slope models and are used for household water treatment and access to basic handwashing facilities.

<u>Modeling of household water treatment</u>

$$Y_{ij} = \beta_0 + \beta_1 X_{1ij} + \sum_{k=2}^{6} \beta_k X_{kj} + \mu_j + \varepsilon_{ij}$$

where Y_{ij} is the proportion of household water treatment (for each water treatment category) for year *i* and country j , β_0 is the overall mean intercept of Y across countries, β_1 is the regression coefficient for the predictor year X_{1ij} (defined at level 1: individual observations-level), β_k are the regression coefficients for the regional covariates X_{kj} (defined at level 2: country-level), μ_j are the residuals on the country-level (group-level residuals) and ε_{ij} are the residuals at the level of the observations (individual observations-level residuals).

<u>Modeling of basic handwashing facility presence</u>

$$\text{logit}(Y_{ij}) = \beta_0 + \beta_1 X_{1ij} + \sum_{k=2}^{6} \beta_k X_{kj} + \sum_{l=7}^{9} \beta_l X_{lj} + \mu_j + \varepsilon_{ij}$$

where logit(Y_{ij}) is the proportion of presence of a designated handwashing facility on the logit scale for year *i* and country j , β_0 is the overall mean intercept of Y across countries, β_1 is the regression coefficient for the predictor year X_{1ij} (defined at level 1: individual observations-level), β_k are the regression coefficients for the regional covariates X_{kj} (defined at level 2: country-level), β_l are the regression coefficients for the income level covariates X_{lj} (defined at level 2), μ_j are the residuals on the country-level (group-level residuals) and ε_{ij} are the residuals at the level of the observations (individual observations-level residuals).

Regional grouping for modelling of household water treatment follow m49 classification and divided as: Africa, Americas, Asia, Europe and Oceania.

Income level covariates include: low income, lower-middle income, higher-middle income.

3. Estimation of safely managed drinking water adjusted for household level and point of use quality

Background: Exposure to safely managed drinking water at the household level is the counterfactual minimum risk exposure level in the analysis of drinking water-attributable diarrhoea. Estimates for population exposure to safely managed drinking water by country for the year 2019 are derived using multilevel modeling of JMP data, which calculate safely managed drinking water services at the level of the urban and rural domains. These estimates of safely managed drinking water were adjusted because: i) the JMP uses data on microbiological quality of drinking water at the point of collection with no consideration of changes in water quality at point of use (e.g., through unhygienic household storage), and ii) the JMP safely managed drinking water estimates are based on the minimum of quality, availability and accessibility at urban and rural levels for each country, since data on these factors typically comes from different sources and cannot be integrated at the household level.

Methods: We adjusted the modeled exposure estimates of safely managed drinking water by integrating nationally-representative information on deterioration of water quality between point-of-collection and point-of-use and on safely managed drinking water access at the household-level from 38 nationally-representative household surveys. We used data from these household surveys (MICS and other national surveys, available through the JMP) that collected data on all three elements of safely managed drinking water: (i) accessibility on premises, (ii) availability when needed, and (iii) freedom from faecal contamination at (a) both the urban/rural domain levels and household levels and (b) point-of-collection water quality as well as point of use water quality[3]. The 38 household surveys cover 17 countries from the African region, 6 countries from the region of the Americas, 5 countries from the Eastern Mediterranean Region, 2 countries from the European region, 2 countries from the South-East Asian region and 6 countries from the Western Pacific region and comprise 9 low-income, 18 lower middle-income and 11 upper middle-income countries. Data from these surveys can be used to explore the relationship between safely managed drinking water categorized at the urban and rural domain level with no faecal contamination at the point of collection (smdw_domain_poc) and safely managed drinking water categorized at the household level with no faecal contamination at the point of use (smdw_hh_pou). smdw_hh_pou can then be modelled for all low- and middle-income countries for which a modeled estimate for smdw_domain_poc is available for the year 2019.

We analysed smdw_hh_pou as a function of smdw_domain_poc as main predictor in a linear regression model. As both the dependent variable and the main predictor are proportions, they were logit-transformed to account for their distribution between 0 and 1.

The following additional covariates were explored in the linear model:

- a squared term of the main predictor smdw_domain_poc.
- different regional covariates: the six WHO regions (see section 3, indicator variables) and region m49, level 1 (5 indicator variables, used for SDG reporting)
- country income status (low income, lower-middle income, upper-middle income, indicator variables)
- per capita GDP (gross domestic product) for 2019 (continuous variable, natural log transformed)

The final model was chosen based on statistical tests for significance (for covariates), the value of the adjusted R^2 and the Akaike's and Bayesian Information Criterion (AIC and BIC).

For countries with an actual value for smdw_hh_pou the modeled value of smdw_domain_poc based on JMP estimates was multiplied with the ratio of smdw_hh_pou / smdw_domain_poc. For 98 LMIC with no value for smdw_hh_pou predictions of this variable were generated through the linear regression model with CIs derived using standard errors of the forecast.

Model diagnostics were performed by plotting residuals against the linear predictions and Q-Q-plots.

Results: smdw_hh_pou shows strong positive linear correlations with smdw_domain_poc (Pearson correlations coeeficients 0.9 for both urban and rural datasets.

The final model for the urban dataset consisted of a linear model including smdw_domain_poc (logit transformed) and log per capita GDP as continuous variables and smdw_domain_poc (logit transformed) plus its squared term as predictor for the rural dataset (Table S1.2).

Table S.2: Model summary for urban/rural model

**Urban model**	**Rural model**
Dependent variable: smdw_hh_pou (logit transformed)	Dependent variable: smdw_hh_pou (logit transformed)
Smdw_domain_poc (logit): p: <0.001	Smdw_domain_poc (logit): p: 003
GDP capita (2019, ln): p=0.07	Smdw_domain_poc squared (of logit smdw_domain): 0.02
R^2: 0.76	R^2: 0.81

The mean difference between the modeled and actual values for smdw_hh_pou for those countries (n=38) where this data was available was 0.9 percentage points for the urban and 0.5 percentage points for the rural dataset.

After the described adjustment, we estimate that 37.9% (29.1%, 49.9%) of the population in LMICs used safely managed drinking water at the household level. This compares to 56.3% (50.8%, 61.8%) without the adjustment.

4. Uncertainty estimates at country, regional and global levels

We estimated uncertainty intervals for the PAFs and attributable disease burden (deaths and DALYs) at the country level with Monte Carlo simulations, taking account of uncertainty of the exposure estimates, as well as the uncertainties of the matching exposure-response relationships, and the total disease burden. For the various exposure levels and exposure-response relationships, we drew random samples of size 10,000 for every country assuming normal distributions after logit or log transformation with the standard error of the logit exposure estimate or the log relative risk as standard deviation. Each randomly generated PAF by country was multiplied with a random draw of the overall disease burden for the condition of interest (diarrhoea, ARI, protein-energy malnutrition, soil-transmitted helminthiasis) assuming a log-normal distribution with the point estimate as the mean, to correct for skewness. The latter allows for the mean of the Monte Carlo result distribution being equal to the result calculated with the point estimates directly. Confidence intervals for the PAFs and attributable disease burdens were set as the difference between the 2.5th and 97.5th percentile.

5. WASH-attributable burden of malnutrition

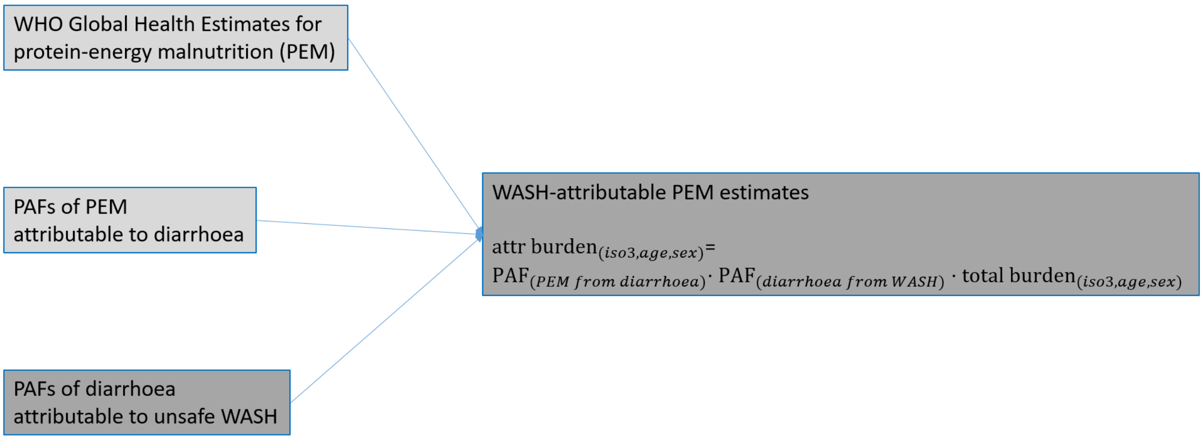

Figure S.1: Conceptual framework for calculating the WASH-attributable burden of undernutrition mediated via the WASH-attributable burden of diarrhoea; light grey: external data inputs, dark grey: estimated in this work

WHO total disease burden figures on malnutrition include ICD 10 codes E40-E46 which are not further disaggregated into wasting, stunting or underweight (Table S.3).

Table S.3: Composition of protein-energy malnutrition as reported by the WHO Global Health Observatory (GHO)

ICD 10- codes (version 2019)	
E40	Kwashiorkor
E41	Nutritional marasmus
E42	Marasmic kwashiorkor
E43	Unspecified severe protein-energy malnutrition
E44	Protein-energy malnutrition of moderate and mild degree
E45	Retarded development following protein-energy malnutrition
E46	Unspecified protein-energy malnutrition

Source: ICD-10, version: 2019, https://icd.who.int/browse10/2019/en#/E40

ICD-10 codes included for diarrhoea, ARI and soil-transmitted helminthiasis are listed in Tables S.6-S.8.

6. Sources of exposure, exposure-response and disease data by outcome

Table S.4: Sources of exposure, exposure-response and disease data by outcome

Health outcome	Source of exposure data	Source of exposure-response relationship	Source of disease data
diarrhoea	JMP household survey database	Wolf et al. 2022[4]	WHO Global Health Estimates[2]
Acute respiratory infections	JMP household survey database	Ross et al. 2023[5]	WHO Global Health Estimates[2]
Protein energy malnutrition	JMP household survey database	Troeger et al. 2018[6]<br><br>Wolf et al. 2022[4]	WHO Global Health Estimates[2]
Soil-transmitted helminth infections	NA	NA	WHO Global Health Estimates[2]

Table S.5: Exposure response functions linking WASH and diarrhoea[4] and hygiene and acute respiratory infections

	diarrhoea	ARI
WATER		
Basic drinking water*	0·81 (0·70, 0·94)	
Basic, on premises, higher water quality*	**0·48 (0·26, 0·87)**	
POU chlorination*	0·66 (0·56, 0·77)	
POU solar treatment*	0·63 (0·50, 0·80)	
POU filtration*	0·50 (0·41, 0·60)	
SANITATION		
Basic sanitation, without sewer connection#	0·79 (0·61, 1·03)	
Basic sanitation with sewer connection#	**0·53 (0·30, 0·93)**	
HYGIENE		
Handwashing with soap§	**0.7 (0.64, 0.76)**	**0.83 (0.76, 0.90)**

POU: point of use, * compared against surface unimproved or limited drinking water; # compared against open defecation, unimproved or limited sanitation; § comparison is handwashing with soap promotion (with or without provision of handwashing infrastructure against no handwashing with soap promotion; bold: relative risk for minimum risk exposure level in this analysis

Table S.6: Composition of diarrhoea as reported by the WHO Global Health Observatory (GHO)

ICD 10- codes (version 2019)	
A00	Cholera
A01	Typhoid and paratyphoid fevers
A03	Shigellosis
A04	Other bacterial intestinal infections
A06	Amoebiasis
A07	Other protozoal intestinal diseases
A08	Viral and other specified intestinal infections
A09	Other gastroenteritis and colitis of infectious and unspecified origin

Source: ICD-10, version: 2019, https://icd.who.int/browse10/2019/en#/A00

Table S.7: Composition of acute respiratory infections as reported by the WHO Global Health Observatory (GHO)

ICD 10- codes (version 2019)	
H65	Nonsuppurative otitis media
H66	Suppurative and unspecified otitis media
J00	Acute nasopharyngitis [common cold]
J01	Acute sinusitis
J02	Acute pharyngitis
J03	Acute tonsillitis
J04	Acute laryngitis and tracheitis
J05	Acute obstructive laryngitis [croup] and epiglottitis
J06	Acute upper respiratory infections of multiple and unspecified sites
J09	Influenza due to identified zoonotic or pandemic influenza virus
J10	Influenza due to identified seasonal influenza virus
J11	Influenza, virus not identified
J12	Viral pneumonia, not elsewhere classified
J13	Pneumonia due to Streptococcus pneumoniae
J14	Pneumonia due to Haemophilus influenzae
J15	Bacterial pneumonia, not elsewhere classified
J16	Pneumonia due to other infectious organisms, not elsewhere classified
J17	Pneumonia in diseases classified elsewhere
J18	Pneumonia, organism unspecified
J20	Acute bronchitis
J21	Acute bronchiolitis
J22	Unspecified acute lower respiratory infection

Source: ICD-10, version: 2019, https://icd.who.int/browse10/2019/en#/H65; https://icd.who.int/browse10/2019/en#/J00; https://icd.who.int/browse10/2019/en#/J09-J18; https://icd.who.int/browse10/2019/en#/J20-J22

Table S.8: Composition of soil-transmitted helminthiasis (intestinal nematode infections) as reported by the WHO Global Health Observatory (GHO)

ICD 10- codes (version 2019)	
B76	Hookworm diseases
B77	Ascariasis
B79	Trichuriasis

Source: ICD-10, version: 2019, https://icd.who.int/browse10/2019/en#/B76

Composition of protein-energy malnutrition as reported by the GHO is listed in Table S.3.

7. Exposure estimates

Table S.9: Distribution of the population to exposure levels of drinking water, by region, for 2019

	Percentage of population (95% CI) using..		
Region	safely managed drinking water sources at household level plus 0 *E-coli* at POU	basic drinking water sources	surface, unimproved or limited drinking water
Sub-Saharan Africa, LMIC	8.8 (6.9, 11.2)	65.6 (63.5, 67.7)	34.4 (32.3, 36.5)
America, LMIC	59.3 (42.3, 74.3)	96.7 (94.1, 98.2)	3.3 (1.8, 5.9)
Eastern Mediterranean, LMIC	36.2 (28.0, 45.3)	88.7 (86.5, 90.5)	11.3 (9.5, 13.5)
Europe, LMIC	61.9 (44.8, 76.5)	97.0 (95.1, 98.1)	3 (1.9, 4.9)
South-East Asia, LMIC	27.2 (10.7, 53.7)	91.7 (89.0, 93.7)	8.3 (6.3, 11)
Western Pacific, LMIC	55.6 (26.2, 81.6)	92.2 (88.0, 95.0)	7.8 (5, 12)
Total LMIC	37.9 (27.1, 49.9)	88.0 (86.0, 89.7)	12 (10.3, 14)

POU: point of use, LMIC: low- and middle-income countries, CI: confidence interval

Table S.10: Distribution of the population to exposure levels of sanitation, by region, for 2019

	Percentage of population (95% CI) using/practicing..		
Region	Basic sanitation connected to sewer	Basic sanitation	Open defecation, unimproved or limited sanitation
Sub-Saharan Africa, LMIC	7.4 (6.3, 8.7)	35.0 (33.5, 36.4)	65.0 (63.6, 66.5)
America, LMIC	59.4 (54.0, 64.5)	87.1 (85.6, 88.5)	12.9 (11.5, 14.4)
Eastern Mediterranean, LMIC	32.2 (24.5, 41.0)	76.1 (73.2, 78.8)	23.9 (21.2, 26.8)
Europe, LMIC	59.0 (33.9, 80.1)	96.2 (93.4, 97.9)	3.8 (2.1, 6.6)
South-East Asia, LMIC	9.1 (5.7, 14.3)	72.7 (69.3, 75.8)	27.3 (24.2, 30.7)
Western Pacific, LMIC	48.7 (28.2, 69.6)	84.2 (79.6, 88.0)	15.8 (12, 20.4)
Total LMIC	29.7 (23.9, 36.1)	72.7 (70.5, 74.7)	27.3 (25.3, 29.5)

LMIC: low- and middle-income countries, CI: confidence interval

Table S.11: Distribution of the population to exposure levels of hygiene, by region, for 2019

	Percentage of population (95% CI) having access to/practicing..		
Region	Handwashing with soap and water after faecal contact	Basic handwashing facility	No or limited handwashing facility
Sub-Saharan Africa, LMIC	9.2 (5.5, 15.1)	30.0 (27.0, 33.1)	70.0 (66.9, 73.0)
America, LMIC	35.9 (32.6, 39.3)	82.0 (69.2, 90.2)	18.0 (9.8, 30.8)
Eastern Mediterranean, LMIC	21.8 (7.9, 47.6)	71.5 (64.3, 77.7)	28.5 (22.3, 35.7)
Europe, LMIC	24.9 (7.6, 57.2)	95.8 (91.2, 98.0)	4.2 (2, 8.8)
South-East Asia, LMIC	28.4 (24.6, 32.4)	74.4 (65.3, 81.7)	25.6 (18.3, 34.7)
Western Pacific, LMIC	17.3 (15.9, 18.8)	92.3 (74.8, 98.0)	7.7 (2, 25.2)
Total LMIC	22.0 (19.0, 25.4)	73.5 (64.9, 80.6)	26.5 (19.4, 35.1)
Total HIC	50.6 (47.9, 53.3)*	-	-

LMIC: low- and middle-income countries, CI: confidence interval

Table S.12: Distribution of the population to exposure levels of household water treatment, by region, for 2019

	Percentage of population treating their drinking water by..		
Region	filtration or boiling	chlorination	solar radiation
Sub-Saharan Africa, LMIC	8.5	6.8	0.0
America, LMIC	20.8	12.9	0.1
Eastern Mediterranean, LMIC	12.4	2.5	0.1
Europe, LMIC	38.8	1.5	0.1
South-East Asia, LMIC	26.4	2.1	0.1
Western Pacific, LMIC	26.6	1.5	0.1
Total LMIC	26.1	3.7	0.1

LMIC: low- and middle-income countries

8. Details on calculating attributable disease burden estimates following Figure 1 of the main manuscript

For the calculation of the deaths and DALYs attributable to a risk factor, the three following data inputs are needed:

1) the distribution of exposure to (different levels of) the risk factor of interest in the study population (p_j in Figure 1)
2) the exposure-response relationship between (different levels of) the risk factor and the health outcome(s) of interest (RR_j in Figure 1)
3) overall disease statistics, i.e. WHO Global Health Estimates in deaths and DALYs by iso3, outcome, age group and sex (total burden in Figure 1)

Exposure response functions of Table S.5 are combined with the proportion of the population in the respective exposure levels to calculate the PAFs which are then multiplied with respective WHO Global Health Estimates.

9. Regional estimates for WASH-attributable diarrhoea and ARI

Table S.13: Diarrhoea burden attributable to inadequate drinking water by region, 2019

Region	PAF	(95% CI)	Deaths	(95% CI)	DALYs	(95% CI)
Sub-Saharan Africa, LMIC	0.42	(0.38, 0.46)	209,033	(188,883, 232,835)	14,389,811	(12,463,521, 17,073,440)
America, LMIC	0.20	(0.17, 0.27)	4,467	(3,724, 5,871)	341,627	(285,193, 449,556)
Eastern Mediterranean, LMIC	0.35	(0.29, 0.43)	53,940	(41,154, 70,171)	3,453,176	(2,727,407, 4,346,215)
Europe, LMIC	0.13	(0.10, 0.20)	486	(389, 693)	125,942	(93,335, 189,431)

South-East Asia, LMIC	0.29	(0.19, 0.42)	230,821	(145,806, 342786)	8,337,219	(5,429,207, 12,147,332)
Western Pacific, LMIC	0.20	(0.15, 0.31)	6,299	(5,043, 8,320)	603,057	(440,106, 921,424)
Total LMIC	0.35	(0.31, 0.40)	505,046	(418,147, 622,247)	27,250,832	(23,665,814, 31,915,532)

PAF: population attributable fraction, CI: confidence interval, DALYs: disability-adjusted life years, LMIC: low- and middle-income countries; 132 low- and middle-income countries, regional PAF aggregates relate to diarrhoea DALYs

Table S.14: Diarrhoea burden attributable to inadequate sanitation by region, 2019

Region	PAF	(95% CI)	Deaths	(95% CI)	DALYs	(95% CI)
Sub-Saharan Africa, LMIC	0.42	(0.39, 0.47)	211,020	(190,972, 235,531)	14,479,134	(12,593,570, 17,076,920)
America, LMIC	0.24	(0.21, 0.26)	5,273	(4,665, 5,962)	398,091	(353,571, 450519)
Eastern Mediterranean, LMIC	0.33	(0.28, 0.40)	49,749	(39,360, 63,054)	3,261,357	(2,672,534, 3,998,589)
Europe, LMIC	0.19	(0.14, 0.22)	893	(738, 997)	179,133	(128, 689, 209,681)
South-East Asia, LMIC	0.36	(0.30, 0.44)	288,508	(221,719, 372,792)	10,392,260	(8,216,933, 12,968,318)
Western Pacific, LMIC	0.28	(0.23, 0.33)	8,865	(7,633, 10,169)	838,429	(684,272, 994,937)
Total LMIC	0.38	(0.35, 0.41)	564,308	(494,793, 653,164)	29,548,404	(26,550,940, 33,222,352)

PAF: population attributable fraction, CI: confidence interval, DALYs: disability-adjusted life years, LMIC: low- and middle-income countries; 132 low- and middle-income countries, regional PAF aggregates relate to diarrhoea DALYs

Table S.15: Diarrhoea burden attributable to inadequate hygiene by region, 2019

Region	PAF	(95% CI)	Deaths	(95% CI)	DALYs	(95% CI)
Sub-Saharan Africa, LMIC	0.28	(0.23, 0.31)	140,058	(116,147, 155,565)	9,618,663	(7,722,377, 11,285,958)
America, LMIC	0.22	(0.20, 0.25)	4,830	(4,301, 5,487)	371,792	(331,350, 421,931)
Eastern Mediterranean, LMIC	0.25	(0.13, 0.32)	37,489	(15,754, 51,020)	2,487,854	(1,237,616, 3,201,902)
Europe, LMIC	0.25	(0.16, 0.29)	1,014	(679, 1,201)	232,565	(147,527, 276,334)
South-East Asia, LMIC	0.24	(0.18, 0.30)	187,773	(138,350-249,183)	6,762,433	(5,156,334, 8,692,073)
Western Pacific, LMIC	0.26	(0.23, 0.31)	7,331	(6,462, 8,403)	784,762	(671,909, 922,322)
High-income countries	0.18	(0.15, 0.21)	5,290	(4,585, 6,224)	237,797	(208,576, 277,249)
Total	0.26	(0.22, 0.28)	383,786	(322,397, 445,046)	20,495,866	(17,557,832, 22,961,088)

PAF: population attributable fraction, CI: confidence interval, DALYs: disability-adjusted life years, LMIC: low- and middle-income countries; 183 low-, middle- and high-income countries, regional PAF aggregates relate to diarrhoea DALYs

Table S.16: Diarrhoea burden attributable to the cluster of inadequate drinking water, sanitation and hygiene by region, 2019

Region	PAF	(95% CI)	Deaths	(95% CI)	DALYs	(95% CI)
Sub-Saharan Africa, LMIC	0.76	(0.72, 0.79)	377,170	(350,598, 403,477)	25,918,546	(22,931,680, 29,713,824)
America, LMIC	0.52	(0.49, 0.57)	11,569	(10,631, 12,634)	884,798	(814,503, 966,667)
Eastern Mediterranean, LMIC	0.68	(0.60, 0.73)	100,905	(83,842, 118,038)	6,598,741	(5,622,273, 7,510,623)
Europe, LMIC	0.47	(0.40, 0.52)	1,976	(1,690, 2,200)	444,995	(372,649, 493,610)
South-East Asia, LMIC	0.66	(0.57, 0.74)	521,292	(420,992, 639,897)	18,784,186	(15,789,982, 22,156,116)
Western Pacific, LMIC	0.58	(0.52, 0.64)	16,969	(15,290, 18,859)	1,721,318	(1,521,799, 1,952,561)
High-income countries	0.18	(0.15, 0.21)	5,290	(4,585, 6,224)	237,797	(208,576, 277,249)
Total	0.69	(0.65, 0.72)	1,035,170	(929,178, 1,159,750)	54,590,384	(50,033,488, 59,561,844)

PAF: population attributable fraction, CI: confidence interval, DALYs: disability-adjusted life years, LMIC: low- and middle-income countries; 183 low-, middle- and high-income countries, regional PAF aggregates relate to diarrhoea DALYs

Table S.17: Burden of acute respiratory infections attributable to inadequate hygiene by region, 2019

Region	PAF	(95% CI)	Deaths	(95% CI)	DALYs	(95% CI)
Sub-Saharan Africa, LMIC	0.16	(0.13, 0.20)	124,856	(100,818, 150,232)	8,305,466	(6,408,695, 10,602,986)
America, LMIC	0.12	(0.10, 0.15)	29,224	(23,486, 38,290)	772,123	(637,583, 991,966)
Eastern Mediterranean, LMIC	0.14	(0.08, 0.20)	28,295	(16,500, 39,487)	1,928,087	(1,077,763, 2,833,195)
Europe, LMICs	0.14	(0.08, 0.19)	11,819	(6783, 16,337)	442,039	(262,473, 605,155)
South-East Asia, LMIC	0.13	(0.09, 0.19)	71,155	(49,405, 103,679)	3,041,998	(2,079,221, 4,469,792)
Western Pacific, LMIC	0.15	(0.12, 0.20)	51,557	(39.445, 69,130)	1,470,715	(1,133,788, 1,975,246)
High-income countries	0.09	(0.08, 0.12)	38,628	(32,552, 49,372)	617,391	(525,496. 783,303)
Total	0.14	(0.13, 0.17)	355,533	(319,625, 404,826)	16,577,818	(14,256,955, 19,481,266)

PAF: population attributable fraction, CI: confidence interval, DALYs: disability-adjusted life years, LMIC: low- and middle-income countries; 183 low-, middle- and high-income countries, regional PAF aggregates relate to diarrhoea DALYs

10. Comparison of WASH-attributable disease burden between WHO and the IHME Global Burden of Disease (GBD)

Table S.18: WASH-attributable burden of diarrhoea and ARI estimates from the WHO and GBD, over time

Year of estimation	WASH-attributable diarrhoeal deaths estimates (in 1000s) (PAF)		WASH-attributable ARI deaths estimates (in 1000s) (PAF)	
	WHO	GBD*	WHO	GBD*
2010		337 (0.23)		
2012	842 (0.58)			
2013		1,399 (0.95)		
2015		1,766		
2016	829 (0.6)	1,481 (0.89)	370 (0.13)	179 (0.08)
2017		1,422 (0.91)		188 (0.07)
2019	1,035 (0.69)	1,387 (0.91)	356 (0.14)	270 (0.11#)

WASH: drinking water, sanitation, hygiene; PAF: population attributable fraction; GBD: global burden of disease.
* GBD PAFs calculated from attributable deaths divided by all deaths for that condition (both numbers as reported by the respective GBD study
# disease burden for lower respiratory infections

Table S.19: Counterfactual minimum exposure levels for WASH-attributable burden of diarrhoea and ARI estimates, over time and by institution

Year of estimation	Counterfactual minimum exposure level	
risk factor	WHO	GBD*
2010 (GBD)/2012 (WHO)		
drinking water	household water treatment using filters or boiling	improved drinking water facilities
sanitation	improved sanitation facilities	improved sanitation facilities
hygiene	handwashing with soap and water after potential faecal contact	[hygiene not covered as risk factor]
2016		
drinking water	household water treatment using filters or boiling	high quality piped water that has been boiled or filtered before drinking
sanitation	basic sanitation in a community>75% sanitation coverage	sanitation facilities with sewer connection or septic tank
hygiene	handwashing with soap and water after potential faecal contact	access to handwashing station after any contact with excreta including children's excreta
2019		
drinking water	safely managed drinking water (adjusted for safely managed at household level)	high quality piped water that has been boiled or filtered before drinking
sanitation	basic sanitation connected to sewer	sanitation facilities with sewer connection or septic tank
hygiene	handwashing with soap and water after potential faecal contact	access to handwashing station after any contact with excreta including children's excreta

ARI: acute respiratory infections; GBD: global burden of disease, * GBD provides WASH-attributable burden of disease estimates for other years (2013, 2015, 2017), which are not listed here, # hygiene-attributable ARI estimated in 2016 and 2019

11. List of diseases/adverse health outcomes that are linked to inadequate WASH or related risks

Table S.20: List of adverse health outcomes linked to inadequate WASH, unsafe water resource management, and unsafe recreational water use.

Health outcome	Exposure	PAF	attributable deaths (in 1000s)	attributable DALYs (in 1000s)
Diarrhoeal diseases	Inadequate WASH	69%	1,035	54,590
Acute respiratory infections	Inadequate hygiene	14%	356	16,578
Undernutrition	Inadequate WASH	10%	8	825
Soil-transmitted helminthiasis	Inadequate WASH	100%	2	1,942
Trachoma	Inadequate WASH	100%[7,8]	-	194
Schistosomiasis	Inadequate WASH	43%-82%[7,8]	5	700
Malaria	Unsafe water resource management	42%-80%[7,8]	329	26,718
Lymphatic filariasis	Unsafe water resource management, unsafe water bodies	67%[8]	-	1,083
Onchocerciasis	Unsafe water resource management	10%[8]	-	121
Dengue	Unsafe water resource management, unsafe water bodies	95%[8]	29	1,855
Japanese encephalitis	Unsafe water resource management, unsafe water bodies	95%*[8]	no overall disease burden data	no overall disease burden data
Drowning	Unsafe recreational water use	not estimated/ insufficient data		
Arsenicosis	Unsafe drinking water	not estimated/ insufficient data		
Fluorosis	Unsafe drinking water	not estimated/ insufficient data		
Legionellosis	Contaminated aerosols from water sprays, jets or mists	not estimated/ insufficient data		
Leptospirosis	Unsafe water resource management, unsafe water bodies, unsafe drinking water	not estimated/ insufficient data		
Hepatitis A and hepatitis E	Inadequate WASH	not estimated/ insufficient data		
Intoxication from cyanobacteriae	Unsafe water resource management, inadequate drinking water	not estimated/ insufficient data		
Lead poisoning	Unsafe drinking water	not estimated/ insufficient data		
Scabies	Inadequate hygiene, inadequate water supply	not estimated/ insufficient data		

Spinal injury	Unsafe recreational water use	not estimated/ insufficient data		
Poliomyelitis	Inadequate WASH	not estimated/ insufficient data		
Adverse neonatal conditions and maternal outcomes	Inadequate WASH	not estimated/ insufficient data		
Cancer (bladder)	Unsafe drinking water	not estimated/ insufficient data		
Musculoskeletal diseases	Inadequate drinking water	not estimated/ insufficient data		

Table adapted from [9], includes results from [8]; * PAF for environmental risks including inadequate WASH

12. Gather checklist

Table S.21: Checklist of information that should be included in new reports of global health estimates[10]

Item #	Checklist item	Reported on page #
**Objectives and funding**		
**1**	Define the indicator(s), populations (including age, sex, and geographic entities), and time period(s) for which estimates were made.	panel 1; methods, 2nd and 3rd paragraph (above section on comparative risk assessment)
**2**	List the funding sources for the work.	section: Role of the funding source
**Data Inputs**		
For all data inputs from multiple sources that are synthesized as part of the study:		
**3**	Describe how the data were identified and how the data were accessed.	method sections: "Population exposed", "Relative risks linking exposure and health outcome"; overall disease burden statistics and PAFs of malnutrition attributable to diarrhoea in Supplementary tables
**4**	Specify the inclusion and exclusion criteria. Identify all ad-hoc exclusions.	Inclusion of disease outcomes: methods, 3rd paragraph.<br>Risk factor categorization: Methods, 2nd paragraph.
**5**	Provide information on all included data sources and their main characteristics. For each data source used, report reference information or contact name/institution, population represented, data collection method, year(s) of data collection, sex and age range, diagnostic criteria or measurement method, and sample size, as relevant.	Exposure data: Supplementary table, tab: "exposure_input", Supplementary material, section 6<br><br>Exposure-response data: Table 1, Supplementary material, section 6.<br><br>Overall disease statistics: methods section "Population Attributable Fraction (PAF)

		and attributable burden estimates", Supplementary material, section 6; supplementary tables with country disease burden statistics
**6**	Identify and describe any categories of input data that have potentially important biases (e.g., based on characteristics listed in item 5).	methods section "Population exposed", discussion section on limitations
For data inputs that contribute to the analysis but were not synthesized as part of the study:		
7	Describe and give sources for any other data inputs.	NA
For all data inputs:		
**8**	Provide all data inputs in a file format from which data can be efficiently extracted (e.g., a spreadsheet rather than a PDF), including all relevant meta-data listed in item 5. For any data inputs that cannot be shared because of ethical or legal reasons, such as third-party ownership, provide a contact name or the name of the institution that retains the right to the data.	Supplementary tables; table 1
**Data analysis**		
**9**	Provide a conceptual overview of the data analysis method. A diagram may be helpful.	Figure 1 on CRA, Figures 2-4 for WASH analysis frameworks
**10**	Provide a detailed description of all steps of the analysis, including mathematical formulae. This description should cover, as relevant, data cleaning, data pre-processing, data adjustments and weighting of data sources, and mathematical or statistical model(s).	methods and supplementary materials
**11**	Describe how candidate models were evaluated and how the final model(s) were selected.	Supplementary material, section 2 and 3
**12**	Provide the results of an evaluation of model performance, if done, as well as the results of any relevant sensitivity analysis.	NA
**13**	Describe methods for calculating uncertainty of the estimates. State which sources of uncertainty were, and were not, accounted for in the uncertainty analysis.	Supplementary material, section "Uncertainty estimates at country, regional and global levels"
**14**	State how analytic or statistical source code used to generate estimates can be accessed.	Data sharing statement at the end of the manuscript
**Results and Discussion**		
**15**	Provide published estimates in a file format from which data can be efficiently extracted.	Supplementary tables with country burden of disease estimates
**16**	Report a quantitative measure of the uncertainty of the estimates (e.g. uncertainty intervals).	Confidence intervals are provided for all burden of disease estimates.
**17**	Interpret results in light of existing evidence. If updating a previous set of estimates, describe the reasons for changes in estimates.	Discussion, paragraphs 2, 3, 4; Supplementary material section 10
**18**	Discuss limitations of the estimates. Include a discussion of any modelling assumptions or data limitations that affect interpretation of the estimates.	Discussion, paragraphs 5 onwards.

13. Participants of the initial expert meeting on WASH-attributable disease burden estimates

"Consultancy on Assessment of the burden of disease attributable to water, sanitation and hygiene", London School of Hygiene and Tropical Medicine, 8-9 April 2013

- Prof. Jamie Bartram, Gillings School of Global Public Health, University of North Carolina at Chapel Hill, USA

- Prof. Sandy Cairncross, Department of Disease Control, London School of Hygiene and Tropical Medicine, UK
- Prof. Thomas Clasen, Department of Disease Control, London School of Hygiene and Tropical Medicine, UK
- Prof Jack Colford, School of Public Health, Division of Epidemiology, University of California, USA
- Mr Oliver Cumming, Department of Disease Control, London School of Hygiene and Tropical Medicine, UK
- Prof. Valerie Curtis, Department of Disease Control, London School of Hygiene and Tropical Medicine, UK
- Prof. Alan Dangour, Department of Population Health, London School of Hygiene and Tropical Medicine, UK
- Prof. Paul Hunter, Norwich Medical School, University of East Anglia, UK
- Dr Richard Johnston, Water Supply and Treatment, EAWAG, Switzerland
- Dr Lorna Fewtrell, Centre for Research into Environment and Health, Aberystwyth University, UK
- Mr Bruce Gordon, Water Sanitation and Hygiene, Department of Public Health and Environment, World Health Organization
- Ms Jennifer de France, Water Sanitation and Hygiene, Department of Public Health and Environment, World Health Organization
- Dr Annette Prüss-Üstün, Evidence and Policy for Environmental Health, Department of Public Health and Environment, World Health Organization
- Dr Sophie Bonjour, Evidence and Policy for Environmental Health, Department of Public Health and Environment, World Health Organization
- Dr Jennyfer Wolf, Evidence and Policy for Environmental Health, Department of Public Health and Environment, World Health Organization

References

1 World Bank. World Bank Country and Lending Groups. 2021. https://datahelpdesk.worldbank.org/knowledgebase/articles/906519-world-bank-country-and-lending-groups (accessed May 5, 2022).

2 WHO. Global Health Observatory (GHO). WHO. http://www.who.int/gho/en/ (accessed Nov 13, 2017).

3 Bain R, Johnston R, Khan S, Hancioglu A, Slaymaker T. Monitoring Drinking Water Quality in Nationally Representative Household Surveys in Low- and Middle-Income Countries: Cross-Sectional Analysis of 27 Multiple Indicator Cluster Surveys 2014–2020. *Environmental Health Perspectives* 2021; **129**. DOI:10.1289/EHP8459.

4 Wolf J, Hubbard S, Brauer M, *et al.* Effectiveness of interventions to improve drinking water, sanitation, and handwashing with soap on risk of diarrhoeal disease in children in low-income and middle-income settings: a systematic review and meta-analysis. *The Lancet* 2022; **400**: 48–59.

5 Ross I, Bick S, Ayieko P, *et al.* Effectiveness of handwashing with soap for preventing acute respiratory infections in low- and middle-income countries – a systematic review and meta-analysis. *in press* 2023.

6 Troeger C, Colombara DV, Rao PC, *et al.* Global disability-adjusted life-year estimates of long-term health burden and undernutrition attributable to diarrhoeal diseases in children younger than 5 years. *The Lancet Global Health* 2018; **6**: e255–69.

7 Prüss-Ustün A, Wolf J, Bartram J, *et al.* Burden of disease from inadequate water, sanitation and hygiene behaviours for selected adverse health outcomes: an updated analysis with a focus on low- and middle-income countries. *International Journal of Hygiene and Environmental Health* 2019; **222**: 765–77.

8 Prüss-Ustün A, Wolf J, Corvalán C, Bos R, Neira M. Preventing disease through healthy environments: A global assessment of the environmental burden of disease from environmental risks. Geneva, Switzerland: World Health Organization, 2016.

9 WHO. Safer Water, Better Health. Geneva, Switzerland: World Health Organization, 2019.

10 Stevens GA, Alkema L, Black RE, *et al.* Guidelines for accurate and transparent health estimates reporting: the GATHER statement. *PLoS medicine* 2016; **13**: e1002056.
